# Development of a standard evaluation method for microbial UV sensitivity using light-emitting diodes

**DOI:** 10.1016/j.heliyon.2024.e27456

**Published:** 2024-03-08

**Authors:** Kai Ishida, Yushi Onoda, Yasuko Kadomura-Ishikawa, Miharu Nagahashi, Michiyo Yamashita, Shiho Fukushima, Toshihiko Aizawa, Shigeharu Yamauchi, Yasuo Fujikawa, Tomotake Tanaka, Takashi Uebanso, Masatake Akutagawa, Kazuaki Mawatari, Akira Takahashi

**Affiliations:** a Department of Microbial Control, Institute of Biomedical Sciences, Tokushima University Graduate School, Tokushima, Japan; b Department of Preventive Environment and Nutrition, Institute of Biomedical Sciences, Tokushima University Graduate School, Tokushima, Japan; c Nichia Corporation, Tokushima, Japan; d Department of Electrical and Electronic Engineering, Graduate School of Technology, Industrial and Social Sciences, University of Tokushima, Tokushima, Japan

**Keywords:** UV-LEDs, *Escherichia coli*, UV dose, Standard evaluation method, Microorganism inactivation

## Abstract

Ultraviolet (UV) light is an effective disinfection method. In particular, UV light-emitting diodes (UV-LEDs) are expected to have many applications as light sources owing to their compact form factor and wide range of choices of wavelengths. However, the UV sensitivity of microorganisms for each UV wavelength has not been evaluated comprehensively because standard experimental conditions based on LED characteristics have not been established. Therefore, it is necessary to establish a standard evaluation method based on LED characteristics. Here, we developed a new UV-LED device based on strictly controlled irradiation conditions using LEDs for each wavelength (250–365 nm), checked the validity of the device characteristics and evaluated the UV sensitivity of *Escherichia coli* using this new evaluation method. For this new device, we considered accurate irradiance, accurate spectra, irradiance uniformity, accurate dose, beam angle, surrounding material reflections, and sample condition. From our results, the following UV irradiation conditions were established as standard: 1 mW/cm^2^ irradiance, bacterial solution with absorbance value of A_600_ = 0.5 diluted 10 times solution, solution volume of 1 mL, working distance (WD) of 100 mm. In order to compare the effects of irradiation under uniform conditions on inactivation of microorganisms, we assessed inactivation effect of *E. coli* by LED irradiation at each wavelength using the U280 LED as a standard wavelength. The inactivation effect for U280 LED irradiation was −0.95 ± 0.21 log at a dose of 4 mJ/cm^2^. Under this condition of dose, our results showed a high wavelength dependence of the inactivation effect at each UV wavelength peaking at 267 nm. Our study showed that this irradiation system was validated for the standard UV irradiation system and could be contributed to the establishment of food and water hygiene control methods and the development of equipment for the prevention of infectious diseases.

## Highlights

• Ultraviolet light-emitting diodes (UV-LEDs) is an effective method of inactivating microorganisms.
• A new UV-LED device based strictly on the optical characteristics of LEDs has developed.
• Standardized irradiation system and evaluation method for assessing UV sensitivity of microorganisms were established.
• This evaluation method using the standardized irradiation system will be useful for assessment of the UV sensitivity of microorganisms in air, food, water, and for material samples.

## 1 Introduction

Pandemic-associated infectious diseases of bacteria, viruses, and fungi, such as *Mycobacterium tuberculosis*, *Vibrio cholera*, *Candida auris*, influenza A virus (H1N1) and severe acute respiratory syndrome coronavirus 2, are problematic because these microorganisms are present in many human livings spaces [[1], [2], [3], [4], [5], [6]]. Infection routes include exposure to airborne droplets, contact with droplet contaminants, ingestion of contaminated food and drinking water, and exposure to water in the environment [[7], [8], [9], [10], [11]]. Therefore, novel and highly effective inactivation methods for a variety of microorganisms are required in human living spaces. Inactivation by chemicals or heat treatment is a common inactivation method. However, there have been some disadvantages to inactivation by chemicals, such as residual chemicals in human space and in food and beverages, and denaturation of components in food and beverages to inactivation by heat treatment. Whereas, photocatalytic inactivation and ultra-violet (UV) inactivation have been attracting attention in recent years. Photocatalysis is an inactivation method that utilizes a chemical reaction when a catalyst is combined with visible or UV light, and has advantages in terms of cost, chemical stability, and environmental friendliness. Photocatalysts are being actively researched mainly in the field of water disinfection because they can decompose contaminants as well as inactivate microorganisms in water [[12], [13], [14], [15], [16], [17], [18]]. On the other hand, UV inactivation, which inactivates microorganisms with UV light itself, is attracting attention in a wide range of fields, including not only water but also human living spaces and foods, because it does not produce any residue after inactivation and does not alter the quality of the object.

UV inactivation is an inactivating method for pathogenic microorganisms that involves irradiation of objects with electromagnetic waves of 10–400 nm, and was approved by the US Food and Drug Administration in 2000 as an effective method for inactivating pathogens in food, water, and beverages [19]. Inactivation of microorganisms using UV light has been actively studied with mercury lamps such as low-pressure mercury lamp and medium pressure mercury lamp [[20], [21], [22]], tunable wavelength lasers [[23], [24], [25]], and light emitting diodes (LEDs). In recent years, UV-LEDs have attracted attention for utilization in a broad field such as water, food and room because the broad range of wavelengths; the compact form factor, providing design flexibility; the longevity of the effect; the adjustable output, which can be controlled by driving current; and the environmental friendliness of the approach, without production of hazardous wastes, such as mercury [[26], [27], [28]]. Evaluations of UV sensitivity is based on how the object changes in response to UV dose, which is the product of irradiance and irradiation time; therefore, it is necessary to accurately determine the irradiance of the target surface and set an appropriate irradiation time. The irradiance on the target surface must be stable, the irradiance distribution must be uniform, and the irradiation time must be accurately controlled. Previous studies have primarily employed mercury lamps, and experimental conditions for mercury lamps have already been established [22]. In standard protocols, the collimation of the beam for accurate irradiance measurement, the irradiance uniformity in the Petri dish, the stability of irradiance and transmittance of the bacterial solution, and the reflection of the beam on the surface of the bacterial solution are described [23]. However, in previous studies using mercury lamps or lasers, the irradiance for each wavelength was not standardized when evaluating UV sensitivity among multiple wavelengths, and the discussion was based on the total integrated dose by adjusting the irradiation time according to the irradiance. For example, in a study comparing the effects of multiple wavelengths using medium pressure mercury lamps and bandpass filters, the irradiance for each wavelength was different in the range of 0.039 mW/cm^2^ to 0.183 mW/cm^2^, and the experiments were conducted at low irradiance due to lamp power issues [21]. A study comparing the effects of multiple wavelengths using a tunable wavelength laser similarly found that the irradiance varied from 10 μW/cm^2^ to 300 μW/cm^2^ for each wavelength [24]. Because the irradiance of LEDs can be adjusted by altering the driving current, they are very useful devices for evaluating UV sensitivity at multiple wavelengths under uniform and constant irradiance conditions. However, one protocol for LED irradiation conditions have been described in a report of water disinfection evaluation [29]; no protocols have been published establishing experimental conditions for UV sensitivity evaluation with UV-LEDs based strictly on the optical characteristics of LEDs, such as the temperature versus peak wavelength characteristics, temperature versus relative radiation flux characteristics, and power supply characteristics, such as overshoot and time delay. Therefore, it is necessary to develop irradiation devices that take into account the characteristics of LED and power supply to accurately assess the sensitivity of microorganisms to UV-LEDs, and to unify evaluation methods using these devices.

In this study, we prepared LEDs of various wavelengths (250–365 nm), developed a new UV irradiation system based strictly on irradiation conditions using LEDs of each wavelength. In addition to factors such as irradiance of the irradiated surface, uniformity of irradiance, and beam angle, the design of this system takes into account factors such as electrical temperature characteristics of the LED, response characteristics of the power supply, irradiation time control, reflection of surrounding materials, and sample temperature, making it possible to accurately evaluate the UV sensitivity of the sample. In addition, by using LEDs as the light source, the system can accurately evaluate the effects of multiple wavelengths of UV at a constant irradiance of 1 mW/cm^2^. Using this system, the UV sensitivity of *Escherichia coli* was evaluated to verify its validity as a standardized UV-LED irradiation system. The evaluation method of UV sensitivity using the irradiation system developed in this study will be useful for assessment of the UV sensitivity of microorganisms in air, food, water, and for material samples.

## 2 Material and methods

### 2.1 Configuration of the UV irradiation system

**Fig. 1:**
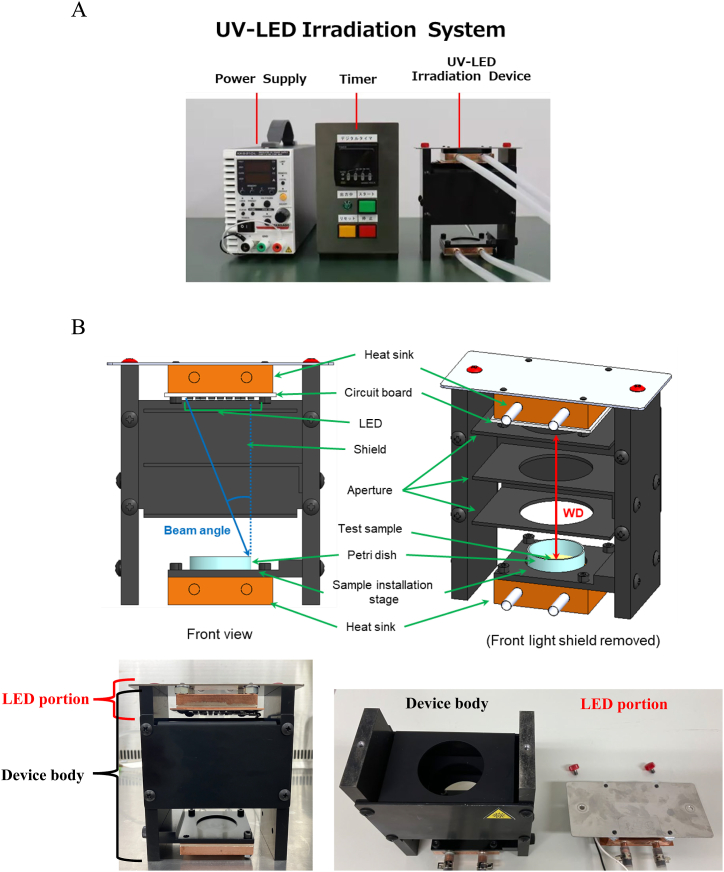
Schematic view of the UV-LED irradiation system. (A) The UV-LED irradiation system consists of a power supply, timer and UV-LED irradiation device. (B) The inner structure of the UV-LED irradiation device, including the front view and top view of the device. The device consists of an LED portion, which contains an LEDs, circuit board and a heat sink, and a device body with apertures for the collimating structure and a heat sink at the sample installation stage. The device body is coated with a low-reflection material to prevent stray light caused by UV reflections inside the device.

A new UV-LED irradiation system was developed based strictly on the temperature and electrical characteristics of the LEDs, irradiance on the sample surface, irradiance uniformity, irradiation time, beam angle, reflection of surrounding materials, and sample temperature (Fig. 1). The UV irradiation system consisted of power supply, timer, and the UV irradiation device. And the device consisted of an LED portion, which contained LEDs, a circuit board, and a heat sink, and a device body with apertures to remove beams with large angles and a heat sink at the sample installation stage. The temperature increase in the LEDs was suppressed by connecting the cooling water flow path inside of the heat sink to a chiller. The inner surface of device body was coated with a low-reflection material to prevent stray light caused by UV reflections from affecting the device, and a heat sink was connected to the sample installation stage to enable temperature control of the sample. In addition, to investigate the effect of irradiance uniformity and beam angle on the inactivation measurement, several device bodies with different working distances (WDs) were prepared and designed so that the working distance could be easily changed by replacing the device bodies, because the distance between UV-LEDs and sample surface largely affects irradiance uniformity and beam angle.

### 2.2 UV radiation source

In this study, UV-LEDs of 13 wavelengths rank (U250, U254, U257, U260, U263, U267, U270, U275, U280, U290, U300, U308, and U365; Nichia Corporation, Tokushima, Japan; Table 1) were prepared. By using small LEDs, optimizing the arrangement of LEDs on the circuit board, and optimizing the number of LEDs to be mounted, we designed a UV-LED radiation device that could provide irradiation beams with high irradiance uniformity. In order to accurately compare the UV sensitivity at each wavelength, the irradiance of the UV light at the surface of the sample solution was standardized to 1 mW/cm^2^ (0.5 mW/cm^2^ for U250, 1.5 mW/cm^2^ for U290, and 18 mW/cm^2^ for U365).

**Table 1:** Peak wavelength and rank of the LED.

LED rank	U250	U254	U257	U260	U263	U267	U270	U275	U280	U290	U300	U308	U365
Peak wavelength (nm)	250.8	253.3	255.7	260.3	263.0	266.8	269.3	274.0	281.3	289.7	300.2	307.5	367.0
Irradiance setting (mW/cm^2^)	0.5	1.0	1.0	1.0	1.0	1.0	1.0	1.0	1.0	1.5	1.0	1.0	18

### 2.3 Irradiance measurement

The irradiance was measured using a radiometer (in-house radiometer, Nichia Corporation). This radiometer was calibrated and adjusted using a standard photodetector calibrated by Japan Calibration Service System. Because the irradiance measurement is greatly affected by the photosensitive area of the photodetector, a photodetector with a sufficiently small photosensitive area (φ1 mm) was mounted. Additionally, the light contained not only vertical light but also angled light. Because this angled light caused errors in irradiance measurements owing to cosine errors, it was necessary to use a radiometer that corrected for those errors, or to develop a collimated UV irradiation system. In this study, along with the removal of beams with large angles by apertures, accurate irradiance measurements were made using a radiometer with a spectral function and cosine correction that was very close to an ideal cosine curve (Fig. S1). In addition, based on the effect of the decrease in output irradiation with LED use, an inspection was conducted to control the irradiance to an appropriate level before conducting every experiment. The dose of radiant energy per second irradiated to the sample at each test wavelength measured by this method is expressed by the word "irradiance" in units of mW/cm^2^, and the dose of radiant energy irradiated to the sample is expressed by the word "dose" in units of mJ/cm^2^, given by *E × t*, where *E* is the irradiance, and *t* is the exposure time in seconds [22].

### 2.4 Spectrum measurement

The wavelengths of individual LEDs differ slightly owing to individual variations, even among those of the same manufacturing rank. And also, LEDs have a characteristic relationship between temperature and peak wavelength such that the wavelength changes depending on the temperature of the LED chip, a feature known as the junction temperature (Tj), and have a characteristic relationship between driving current and peak wavelength such that the wavelength changes depending on the driving current. Therefore, when irradiation is performed using UV-LEDs, the wavelength of the entire LED system may differ from the catalog wavelength of each LED owing to individual variations in LED wavelengths and the increase in the Tj caused by heat dissipation of the mounted LEDs. Accordingly, it is necessary not only to suppress the increase in LED temperature using cooling but also to measure the characteristics of each LED at each wavelength separately and consider the effects of those variations during operation.

**Fig. 2:**
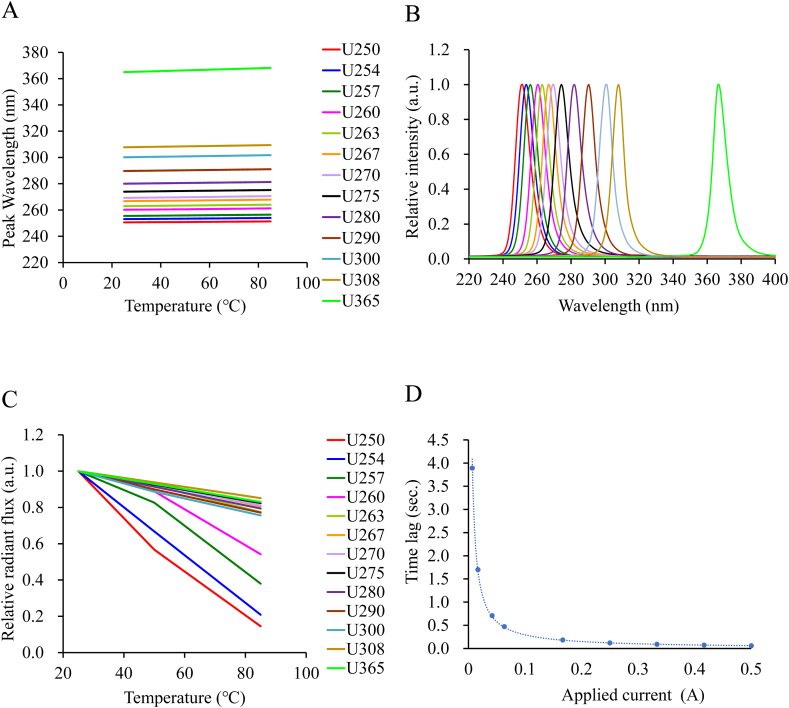
Characteristics of the UV-LEDs irradiation device. (A) Peak wavelength variation with temperature for each LED (U250, U254, U257, U260, U263, U267, U270, U275, U280, U290, U300, U308, U365) in this study. (B) Measurement of LED spectrums at each wavelength of LEDs on the circuit bord in this study. (C) Relative radiant flux variation with temperature for each LED. (D) Plot of applied current versus power supply time lag.

In the UV-LED irradiation system developed in this study, the temperature versus peak wavelength characteristic of LEDs was measured at each wavelength, and we confirmed the very slight wavelength variation within the range of junction temperature (Tj ≤ 46.5) occurring in this system (Fig. 2A). We also measured the overall wavelengths of the entire system by measuring the total spectra with several LEDs mounted on the circuit board (Fig. 2B).

### 2.5 Control of irradiance and electrical and temperature characteristics of LEDs

LEDs can decrease radiation flux in response to their junction temperature. This feature is called the temperature versus relative radiant flux characteristic, which is derived from the epi-design and device structure design. Because these designs are different for each wavelength, the temperature versus relative radiation flux characteristic is also different for each wavelength.

The temperature versus relative radiant flux characteristic of the LEDs for each wavelength used in this study was measured to assess their respective characteristics (Fig. 2C). We then calculated the Tj of the LED when the LED is driven from the transient heat measurement, and simulated the irradiance based on the Tj. Based on these data, we confirmed that the difference between the irradiance measurement using a radiometer and the simulation was within ±10%. In the case of the 250 nm LED, which had the high LED heat dissipation, Tj increased to 46.5 °C within 1 s and remained stable at that temperature thereafter (Fig. S2A).

### 2.6 Power supply characteristics

The overshoot is the waveform that exceeds the baseline of the steady state value at the rising edge of the current waveform, which occurs immediately at the startup in DC power supplies. In this study, The DC power supply (KX-S-210-L; Takasago, Ltd.) provided a current of 2.4 A to the LED in constant current mode. The light intensity was monitored with a photodiode (S2281; Hamamatsu Photonics K.K). The photocurrent of the photodiode was converted to a voltage using an oscilloscope (DL1740; YDK Technologies Co., Ltd.) with input impedance of 50 Ω. As a result, the excess current due to overshooting was approximately 3% of the total current when irradiated for 2 s (Fig. S2B). These data indicated that the percentage of excess current due to overshooting was negligibly small.

In addition, LEDs have the advantage that their output can be controlled by current. However, because of the characteristics of the DC power supply, a time lag occurs between the time the power is turned on and the time the current actually flows, which may affect the total amount of beam emitted from the UV-LED. Additionally, the time lag varies depending on the current value. Furthermore, electrical energy efficiency of LEDs of each wavelength are different, so that it is necessary to apply a current corresponding to the electrical energy efficiency of each wavelength of LEDs in order to make LEDs with different wavelength emit light at the same irradiance. So, variations in the power supply time lag caused by this difference in applied current can also affect comparisons among multiple wavelengths.

In this study, we measured the time lag of a DC power supply at each current value and plotted the results (Fig. 2D). According to this plot, the time lag corresponding to the applied current of each wavelength of LEDs were added to the irradiation time to compare the inactivation effect of LEDs of different wavelengths with accurate totalized dose. In general, there was a slight error between the current value shown on the display screen and the current value actually flowing in the power supply. In this study, the current value to be applied to each LED was not the current value indicated on the screen of the DC power supply, but the actual current value flowing, measured with a current probe.

### 2.7 Irradiation time control

In order to accurately control the LED irradiation time, a DC power supply and a timer with a time resolution of 0.1 s were connected, and the LED ON/OFF was designed to be controlled by the timer. This enabled us to compare the inactivation effects of LEDs of different wavelengths with accurate integrated dose.

### 2.8 Influence of light reflection

When UV light is irradiated onto a bacterial solution, stray light is generated by reflection and scattering of the beam inside the device or sample installation stage directly under the Petri dish. When this unexpected beam irradiates the sample, errors in the UV sensitivity evaluation could occur. In this study, the entire surface of each device body was coated with a low-reflection material to prevent stray light (Fig. 1A). In addition, to investigate whether the irradiance error caused by reflection affected the UV sensitivity evaluation, we prepared two types of sample installation stages (with or without a low-reflection coating) and investigated the effects of the reflected light from stage material directly under the Petri dish on the UV sensitivity evaluation.

### 2.9 Beam angle

When UV light is irradiated onto a bacterial solution, a portion of the UV light may be reflected back into the air due to the difference in refractive index between the air and the sample solution. The percentage of beam lost by this reflection can be calculated as a function of the refractive index and beam angle of the air and the bacteria solution, and if the bacterial solution is water-based, the loss can be kept within 2% if the beam is within a 20° angle. In this study, device bodies with working distances of 50, 100, and 200 mm, were produced, and irradiation conditions were assessed with different percentages of light within 20° by adjusting the distance between the LED and the bacterial solution using device bodies with different WDs. Each device body was coated with low-reflection material on all surfaces and equipped with multiple apertures between the LED and the Petri dish to prevent stray light with a large angle from reflection inside the device. The percentages of light within 20° of the beam angles in WDs of the device body were 71.7% at 50 mm, 99.6% at 100 mm, and 100.0% at 200 mm.

### 2.10 Irradiance uniformity

When the irradiance uniformity on the bacterial solution is low, it is a source of error in the evaluation of UV sensitivity because of differences between areas with high irradiance and areas with low irradiance. In this case, when a UV irradiation device consists of multiple UV-LEDs instead of one chip of UV-LED is used, the irradiance at the center of the Petri dish is not always the maximum irradiance. Therefore, in this study, we evaluated irradiance uniformity using two evaluation methods: the ratio of the mean irradiance to the irradiance at the center of the Petri dish and the ratio of the mean irradiance to the maximum irradiance in the Petri dish. In order to irradiate UV light on a Petri dish (φ35 mm), we optimized the arrangement of 36 LEDs on a circuit board suitable for the size of the Petri dish and designed the device to irradiate light with high uniformity. Optical simulations were then performed to confirm the irradiance and irradiance uniformity at WDs of 50, 100, and 200 mm.

### 2.11 Bacterial strain and cultural condition

*E. coli* (ATCC25922) was used as a standard bacterial strain in these experiments. *E. coli* was picked from the glycerol stock (final concentration of glycerol, 15%) at −80 °C and cultured in 1% NaCl-LB broth (1% tryptone, 0.5% yeast extract) for up to 24 h at 37 °C with shaking (170 rpm) until reaching stationary phase after preculturing the day before. Bacterial culture medium was washed three times with d-phosphate buffered saline (D-PBS (−); Fujifilm Wako Chemicals) using a centrifuge at 12,000 rpm for 3 min each wash to remove the culture broth. Subsequently, bacterial suspension was diluted to an absorbance of 600 nm (A_600_) of 0.5 with D-PBS, and the adjusting bacterial solution after a 10-fold dilution (A_600_ = 0.5/10) was used for irradiation experiments.

### 2.12 Irradiation of bacterial suspensions by UV-LEDs

For each experimental condition in the dose response assessment and for comparisons of bacterial inactivation by each of the 13 UV-LED wavelengths, 1 mL prepared bacterial suspension was placed in a 35-mm TC dish (Sarstedt, Nümbrecht, Germany) and exposed to UV irradiation without stirring using the developed UV-LED irradiation device (Fig. 1). The bacterial suspension was irradiated at 0, 2, 4, 6, 8, or 10 mW/cm^2^ using 254-, 267-, 270-, and 280-nm UV-LEDs. Experiments for comparisons of device conditions under UV-LED irradiation were as follows: WD was changed to 50, 100, and 200 mm; stage materials were changed to a low-reflection coating or without a low-reflection coating; suspension volume was changed to 1 or 6 mL; and absorbance of each bacterial suspensions were diluted to A_600_ = 2.5 and 0.5 in addition to A_600_ = 0.5/10.

Here, the transmission spectrum of bacterial solutions from 200 to 700 nm was measured using a UV/Vis-spectrophotometer (Beckman Coulter, Brea, CA, USA). To avoid the possible influence of ambient light such as photoreactivation, the entire process of UV exposure and bacterial inoculation was carried out in a dark place.

### 2.13 Measurement of bacterial inactivation

The irradiated samples were collected in 1.5 mL tubes, diluted with D-PBS, plated (100 μL on 1% NaCl-LB agar), and incubated for 24 h at 37 °C. The number of bacteria was estimated using colony-forming unit (CFU) assays. The inactivating effects of UV-LED irradiation were evaluated using the log10 CFU reduction compared with unirradiated sample, based on the following equation and was defined as “log survival rate”: log survival rate = log10(Nt/N_0_), where Nt is CFU/mL of the UV-irradiated sample and N_0_ is CFU/mL of the sample without UV irradiation.

### 2.14 Statistical analysis

All studies were conducted in four times and on 4 separate days. The data are presented as the mean ± SEM for all experiments. *p* values were calculated using Student's t-test with threshold for significance was set at *p* < 0.05 and *p* < 0.01. As follows: *, *p* < 0.05; **, *p* < 0.01.

## 3 Results and discussion

### 3.1 Calculation of the correction factor for the developed device

To quantitatively evaluate UV irradiation for a sample, several correction factors have been proposed for mercury lamp and UV-LED irradiation [29]. These correction factors include water factor (WF), which is a coefficient of the absorbance and depth of a sample solution; divergence factor (DF), which is a coefficient of the distance from the light source to the sample solution surface and water depth; petri factor (PF), which is a coefficient of irradiance uniformity; reflection factor (RF), which is a coefficient of the beam angle and reflection on the surface of the sample solution; and coefficient of variation (CV), which is the standard deviation of irradiance in the Petri dish divided by the mean irradiance. WF, DF, and PF are considered more appropriate in cases showing values close to 1. Likewise, CV is necessary for values of 6.7% or less to show accurate irradiance uniformity. The correction factor of our developed UV irradiation device was confirmed when the device was irradiated with the U280 UV-LED for the target bacterial samples. Because the device contained a surface light source combining LEDs, the center did not necessarily show the maximum irradiance. Therefore, PF was evaluated using both the irradiance at the center and the maximum irradiance in the Petri dish. In these results, all correction coefficients were very close to 1, and the CV was 2.23% (Table 2). Another important factor is the degree of collimation of the light. There are two terms for receipt of radiation, irradiance and fluence rate [22]. Irradiance (mW/cm^2^) is defined as the total radiant power incident from all directions in a hemispherical solid angle on an infinitesimal element of surface of area d*S* containing the point under consideration divided by d*S*. Fluence rate (mW/cm^2^) is defined as the total radiant power incident from all directions through an infinitesimally small sphere of cross-sectional area d*A*, divided by d*A*. When UV light is collimated, this means that irradiance and fluence rate are virtually the same, and previous study using UV-LEDs have reported that even without a collimating column, these values get closer as the working distance is increased, with irradiance and fluence rate matching over 95% at 100 mm working distance [29]. The developed device has three apertures with a working distance of 100 mm, and 99.6% of the light is contained within a 20° angle. In addition, the radiometer used in this study has a cosine correction that is very close to the ideal cosine curve, so the irradiance confirmed with the radiometer is considered to be very close to the fluence rate. Therefore, the dose of radiant energy expressed as "dose" in this study is very close to the "fluence" used in other studies, which is the product of fluence rate and the exposure time. However, since they are not exactly the same, we use the word "irradiance" to avoid misunderstanding. Taken together, these findings confirmed that UV sensitivity evaluation could be performed with high accuracy using our developed irradiation device.

**Table 2:** The calculation value for each correction factor of UV-LED irradiation.

Correction factor	Water factor	Divergence factor	Petri factor (Ave/Center)	Petri factor (Ave/Max)	Reflection factor	Coefficient of variation
Calculation value	0.984(250 nm) ∼0.992(365 nm) (A_600_ = 0.5/10)	0.990 (Depth = 0.1 cm)	0.985 (φ35mm)	0.984 (φ35mm)	0.97–0.98	2.23% (φ35mm)

### 3.2 Effects of bacterial sample condition on inactivation

**Fig. 3:**
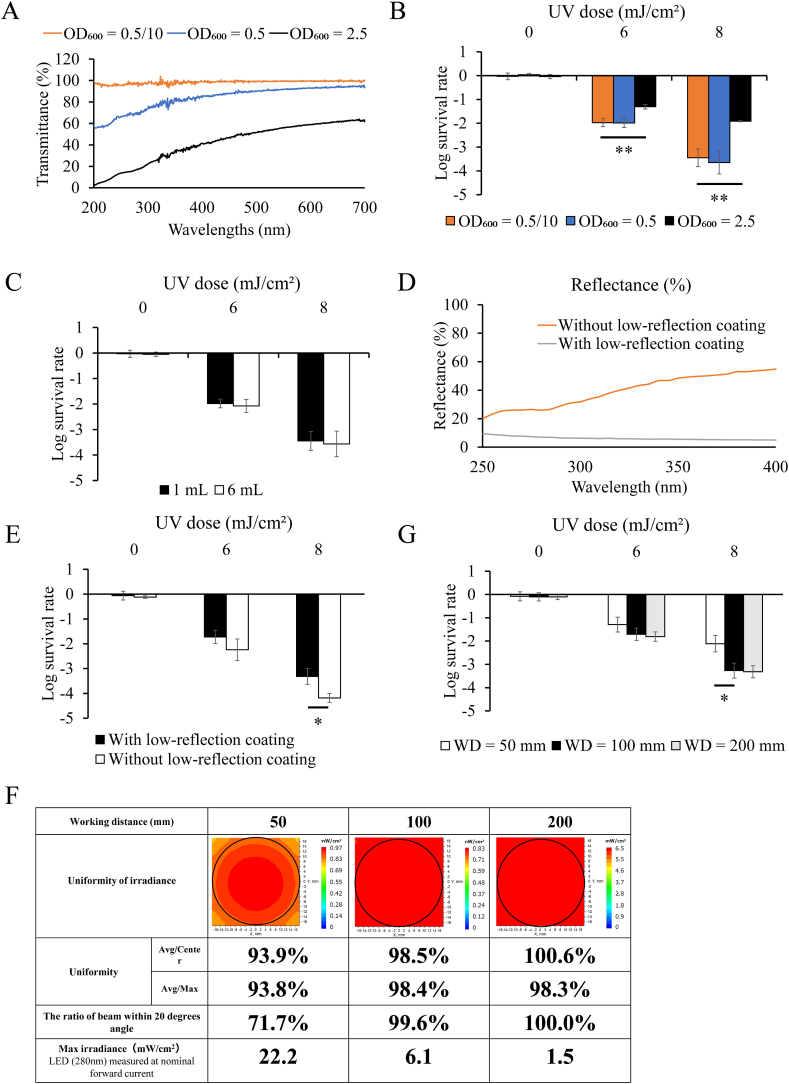
Evaluation of the validity of the developed device. (A) Transmittance through a 1 mm optical path length based on the absorbance of each bacterial solution. (B) Effects of U280 LED irradiation (0, 6, 8 mJ/cm^2^) on inactivation based on the absorbance of the bacterial solution. (A_600_ = 0.5/10, A_600_ = 0.5 and A_600_ = 2.5); n = 4/group. (C) Effects of U280 LED irradiation (0, 6, 8 mJ/cm^2^) on inactivation based on the volume of the bacterial solution. (1 and 6 mL); n = 4/group. (D) Measurement values of reflectance with or without a low-reflection coating on the stage material. (E) Effects of U280 LED irradiation (0, 6, 8 mJ/cm^2^) on inactivation with or without a low-reflection coating on the stage material; n = 4/group. (F) Uniformity of irradiance, the ratio of the beam within 20°, and maximum irradiance at each working distance (WD = 50, 100, 200 mm) (G) Effects of U280 LED irradiation (0, 6, 8 mJ/cm^2^) on inactivation at each working distance (WD = 50, 100, 200 mm); n = 4/group. Each bar indicates the mean concentration. *p < 0.05 and **p < 0.01 (t tests).

To properly evaluate the inactivation effect of irradiation, it is necessary to consider the absorbance and the liquid volume of the bacterial sample. If the absorbance of the bacterial sample is excessively high, the irradiance to the bacteria in the lower layer may not be adequate because the light is absorbed by the bacteria in the upper layer. To evaluate differences in the inactivation effects of absorbance, we measured transmission from 200 to 700 nm and the inactivation effect by U280 irradiation at each absorbance. The absorbance samples were chosen as 10-fold dilution of A_600_ = 0.5 (A_600_ = 0.5/10), A_600_ = 0.5, and A_600_ = 2.5. The transmittance of the sample solution decreased markedly with increasing absorbance, and the measurement values of A_600_ = 0.5/10, A_600_ = 0.5, and A_600_ = 2.5 at 281.3 nm were 97.6, 70.2, and 17.5, respectively (Fig. 3A). Furthermore, the water factors of A_600_ = 0.5/10, A_600_ = 0.5, and A_600_ = 2.5 were 0.985, 0.880, and 0.715, respectively. The results of U280 irradiation indicated similar inactivation for A_600_ = 0.5/10 and A_600_ = 0.5, whereas A_600_ = 2.5 showed significantly lower sensitivity (Fig. 3B). These data indicated that a WF value of 0.9 or higher was required to accurately evaluate UV sensitivity.

When the bacterial solution is deep because of the large volume of solution, the bacteria in the lower layer may not be exposed to an adequate level of irradiation. To evaluate differences in the effects of depth on inactivation, we measured the inactivation effects of U280 irradiation on bacterial solutions of 1 (depth: 0.1 cm) and 6 mL (depth: 0.6 cm). The water factors of 1 and 6 mL were 0.985 and 0.913, respectively, and the divergence factors of 1 and 6 mL were 0.990 and 0.943, respectively. U280 UV-LED irradiation showed similar levels of inactivation for 1- and 6-mL solution volumes (Fig. 3C). These data indicated that a DF of 0.94 or higher did not cause errors in UV sensitivity evaluation for the solution volume. Therefore, we choose an absorbance value of A_600_ = 0.5/10 and a solution volume of 1 mL for UV irradiation in this study because it showed high values for of WF and DF.

### 3.3 Influence of the reflection of the stage material on bacterial inactivation

Reflection of light within the device can be a source of error when evaluating UV sensitivity. To investigate whether irradiance errors caused by reflections affected UV susceptibility evaluation, we prepared stage materials with and without a low-reflection coating on the stage material directly under the Petri dish. Measurements of the reflectance of UV light at wavelengths from 250 to 400 nm for stage materials with and without a low-reflection coating showed lower reflectance values for the stage material with the coating than for that without the coating (Fig. 3D). Accordingly, we irradiated the bacterial solution with the U280 LED at a WD of 100 mm to confirm the inactivation effects of different reflectance levels for the stage material. As a result, a significantly higher inactivation effect was observed when microorganisms placed on the stage without weakly reflective coating were irradiated with 8 mJ/cm^2^ UV light. (Fig. 3E). This result confirmed that the reflection of UV inside the equipment significantly affected the results of the UV sensitivity evaluation. Therefore, stages with low-reflection coating were used in subsequent experiments.

### 3.4 Influence of irradiance uniformity caused by differences in WD on bacterial inactivation

Irradiance uniformity and beam angle are factors that vary depending on the distance between the UV light source and the sample surface. In general, the further the distance, the higher the uniformity and the closer the beam angle to vertical. At the same time, the further the distance, the smaller the percentage of light that hits the Petri dish among the light delivered from the light source. In this study, we used optical simulation to predict the asymmetry of irradiance and the fraction of light within 20° for WDs of 50, 100, and 200 mm. We also assessed the maximum irradiance delivered to the sample when a U280 LED was applied at its rated current. As a result, 100 mm was considered to be appropriate for the evaluation because the WD of 50 mm showed low irradiance uniformity and a low percentage of light within 20°, whereas a WD of 200 mm did not provide sufficient irradiance for UV susceptibility evaluation (Fig. 3F). Next, the UV sensitivity of *E. coli* was evaluated under each of these conditions. The results showed that there were significantly lower log survival rates at 50 mm compared with those at 100 and 200 mm, suggesting that a WD of 100 mm or more was necessary for accurate evaluation of UV susceptibility (Fig. 3G). Based on the above results, a WD of 100 mm was used in subsequent experiments.

### 3.5 Effects of UV irradiation applied using the developed irradiation device on *E. coli*

From the results of our device validity investigations, the following UV irradiation conditions were established: 1 mW/cm^2^ irradiance, absorbance value of A_600_ = 0.5/10, solution volume of 1 mL, low-reflection coating for the stage material, WD of 100 mm. Compared with the conditions used in previous studies of UV irradiation, the conditions for our developed device appeared to yield constant irradiance and high irradiance uniformity, enabled elimination of stray light effects, and did not require shielding effects, such as stirrers [[20], [21], [22], [23], [24], [25],^30^]. In particular, the irradiance uniformity is important for comparing the bactericidal effects among multiple wavelengths. The inactivation mechanism of microorganisms by UV irradiation has been reported to involve the damage of chromophores, such as nucleic acids and proteins, by UV absorption, resulting in alteration of the chemical structure. In addition, the production of reactive oxygen species by UV irradiation also causes damage to intracellular components [31,32]. On the other hand, microorganisms have reactivation mechanisms that involve photoreactivation and dark reactivation to reconstruct nucleic acid and a superoxide dismutase mechanism that removes reactive oxygen species [[33], [34], [35]]. Accordingly, UV-induced damage is calculated as the total amount of actual injury minus the recovery at each irradiation time. Therefore, it is important to use uniform values for accumulated dose, irradiance, and irradiation time in order to compare the effects of different wavelengths on the inactivation and UV sensitivity of different microorganisms. Because the developed device showed uniform irradiance at most wavelengths of UV-LEDs, the device was considered capable of accurately comparing UV sensitivities among multiple wavelengths.

### 3.6 Effects of UV irradiation using the developed irradiation device on *E. coli* inactivation

**Fig. 4:**
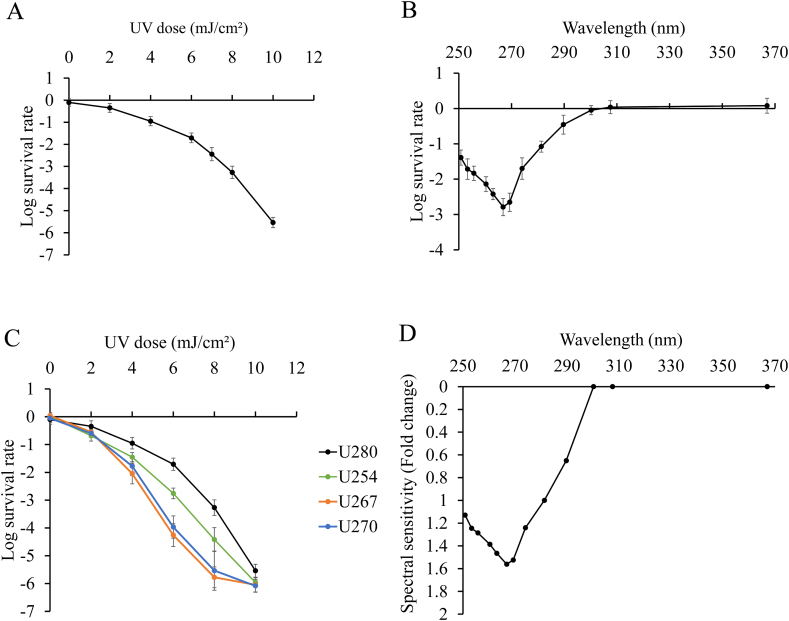
**Effects of the developed UV-LED irradiation device of inactivation of *Escherichia coli.*** (A) Dose response of *E. coli* inactivation following irradiation with; n = 4/group. (B) Effects of irradiation by each LED (U250, U253, U257, U260, U263, U267, U270, U275, U280, U290, U300, U308, and U365) on inactivation of *E. coli* based on −1 log inactivation conditions from U280 UV-LED irradiation; n = 4/group. (C) Dose response of *E. coli* inactivation following irradiation with U267, U270, and U280 LEDs at 0, 2, 4, 6, 8 and 10 mW/cm^2^; n = 4/group. (D) Ratio of the inactivation effects of LEDs (U250, U253, U257, U260, U263, U267, U270, U275, U280, U290, U300, U308, and U365) on *E. coli* calculated based on that of U280.

In order to compare the effects of irradiation under uniform conditions on inactivation of microorganisms, we assessed inactivation effect of *E. coli* by LED irradiation at each wavelength using the U280 LED as a standard wavelength because this wavelength is typically used for inactivation and has a high electrical energy efficiency. First, we analyzed the dose-dependent effects of U280 irradiation on microorganisms. Inactivation of *E. coli* by the U280 LED, as measured by changed in viability, showed a dose-dependent bactericidal effect on dose; the inactivation effect was −0.95 ± 0.21 log at a dose of 4 mJ/cm^2^ (Fig. 4A). Under 4 mJ/cm^2^ of dose, we compared the effects of each UV wavelength on microorganism inactivation. The results showed a high wavelength dependence of the inactivation effect at each UV wavelength; in particular, the inactivation effects were higher at 267–270 nm especially (Fig. 4B). A previous study evaluated the wavelength dependence of *E. coli* inactivation by combining a UV lamp and a monochromator and reported the wavelength dependence peaking at 267.5 nm [36,37]. The tendency of wavelength dependence of inactivation is similar to the results of this study confirming our study. The evaluation system using UV-LEDs was relatively simple, had excellent stability and durability of light irradiation, and was easy to operate. Therefore, it would be suitable for the evaluation of microbial inactivation in relation to UV dose or wavelength. There were some reports which analyzed the UV wavelength dependence of *E. coli* using LEDs with two or three wavelengths [38,39]. For example, a study comparing the inactivation effect of three LEDs with peak wavelengths at 265, 280 and 300 nm reported that UV light of 265 nm wavelength was most effective in inactivating *E. coli* [40]. We evaluated the UV-LED wavelength-dependent inactivation of *E. coli* in with pitch width of a few nanometers using different 13 UV-LEDs with peak wavelengths from 250 nm to 365 nm. This is the first report that the use of UV-LEDs with more finely tuned wavelengths produced detailed differences in the inactivation effect on *E. coli* depending on the wavelength.

In order to evaluate the wavelength dependence of the inactivation in more detail, we examined the dose-dependent inactivation effects of irradiation with U254, U267, U270, and U280 LEDs. Our result showed that differences in inactivation effect from 4 mJ/cm^2^ for U254, U267 and U270 LED irradiation to U280 LED irradiation. Likewise, our result showed that differences in inactivation effect from 6 mJ/cm^2^ for U267 and U270 LED irradiation to U254 LED irradiation (Fig. 4C). In previous studies, inactivation of bacteria by irradiation at 265 nm has been reported to have dramatic effects, potentially owing to nucleic acid damage [31,41]. Thymine, a base contained within nucleic acids, is the simplest nucleobase present in DNA, and previous reports have shown that the peak of the absorption spectrum of thymine is at 265 nm [42]. Because our results also showed that irradiation with the U267 LED showed higher inactivation effects than the other wavelengths, we concluded that irradiation at this wavelength (267 nm) showed a more efficient bactericidal effect owing to the higher absorption efficiency of UV light by nucleic acids. Our results of UV irradiation using LEDs of 13 wavelengths suggested more detailed peak wavelengths for the inactivation effects on *E. coli* in UV-LED irradiation experiment.

The dose-dependent inactivation of *E. coli* reported using low- and medium-pressure mercury lamps, variable wavelength lasers and UV-LED, and it was also showed that there were differences in UV sensitivity among different species of *E. coli* [43]. In addition, even when the same species of *E. coli* was used to evaluate UV sensitivity to low pressure mercury lamps, the results varied widely from report to report. For example, in a study using *E. coli* ATCC 11229 and low-pressure mercury lamps, some reported an inactivation effect of −1 log after 2.5 mJ/cm^2^ irradiation and the other reported an inactivation effect of −1 log after 7 mJ/cm^2^ irradiation [43]. These differences possibly due to differences in experimental methods and environments in each report.

A previous study of ATCC 25922 strains, the same strain used in this study, irradiated UV light with low pressure mercury lamp and collimation tube at relatively precise UV doses reported an average inactivation effect of −4.86 log at 10 mJ/cm^2^ irradiation [44]. In study, irradiation with U254 LED at 10 mJ/cm^2^ resulted in inactivation effect of −5.96 ± 0.6 log, and implicating that U254 LED was more effective than low pressure mercury lamp in their microbial inactivation effect. One hypothesis is that a difference in spectral width affects this difference of results. This is because LEDs emit light with a spectral width of about 10 nm at half maximum, including light with longer wavelengths than 254 nm, which has a high inactivation effect, whereas low-pressure mercury lamps emit light with a narrower width of 254 nm. Another hypothesis is that the difference in the irradiance of the UV light may have affected the results. In fact, a previous study reported an irradiance of 0.1 mW/cm^2^ and an irradiation time 10 times longer than that of the developed device. These differences in the characteristics of LEDs and low-pressure mercury lamps as light sources and/or differences in conditions between experiments might have led to different results when comparing LEDs and low-pressure mercury lamps. However, in terms of the accuracy of irradiation conditions, the device developed in this study is considered more suitable for evaluating the UV sensitivity of microorganisms than any of the previous devices using mercury lamps or tunable wavelength lasers. Using the irradiation device and evaluation method developed in this study as a standard method, it will be possible to rigorously evaluate the UV sensitivity of microorganisms with high reproducibility.

### 3.7 Comparative evaluation method for disinfection efficacy at different UV wavelengths based on required dose

Some calculation methods have been proposed for evaluating UV sensitivity in bacteria using a mercury lamp with a peak wavelength at 253.7 nm as the standard UV irradiation device [36]. These methods read the linear portion from the curve of the log survival rate and UV dose and compare the inclination of the graph of log survival rate versus dose (GF_1ε_(λ)), the ratio of the dose required to achieve a constant log survival rate (GF_2ε_(λ)), and the coefficient calculated using the least squares method at values of mapping the curve at each wavelength to the curve at 253.7 nm after approximating the curve for the log survival rate and UV dose using a quadratic function (GF_3ε_(λ)). We generated calculation formulae for the U280 LED standard with reference to these calculation methods.

(1) GF_1ε_(λ) = k_F0_(λ) / k_F0_(280)

(2) GF_2ε_(λ) = 1/F_0_(λ)_log(n)_ / 1/F_0_(280) _log(n)_

GF_3ε_(λ) was also calculated by changing the reference wavelength from 253.7 to 280 nm. In these formulas, k_F0_ represents a first-order rate constant in the linear portion of the curve, and F_0_ represents the dose (mJ/cm^2^). Using the formulae, we compared the inactivation effects of irradiation with U254, U267, U270, and U280 LEDs irradiation using our newly developed device (Table 3). From these calculation results, the ratio of the inactivation effects of irradiation with the U254, U267, or U270 LED to the U280 LED showed slightly different values depending on the use of the calculation formula and the setting for the log survival rate. In previous studies using mercury lamps and lasers, it was difficult to remove the influence of differences in irradiance from the evaluation method because it was necessary to evaluate the inactivation effect for each wavelength using a uniform dose owing to variations in irradiance at different wavelengths. On the other hand, our new developed device, which showed uniformity of UV light irradiation at different wavelengths, enabled evaluations to be performed without being affected by differences in irradiance. Thus, the calculation results for the inactivation effects of our developed device may be related to variations based on the different calculation formulae.

**Table 3:** The value of irradiation effect by using variety calculation method.

LED rank		U280	U254	U267	U270
GF_1ε_(λ)		1.00	1.31	1.88	1.78
GF_2ε_(λ)	1logs	1.00	1.57	1.84	1.77
	2logs	1.00	1.45	1.66	1.55
	3logs	1.00	1.23	1.56	1.46
GF_3ε_(λ)		1.00	1.09	1.35	1.31
GF_4ε_(λ)		1.00	1.25	1.56	1.52

In applied research on UV inactivation, including the development of UV inactivation equipment, data on the required dose to achieve the goal of inactivation are more useful for the development of applied products than data on the percentage of bacteria inactivated by irradiation to a certain irradiance. In addition, a simpler method to compare the inactivation effects of different wavelengths is required for applications in product development because it is necessary to create dose-dependent inactivation curves for irradiation at each UV wavelength to be compared using typical calculation formulae. Therefore, we propose a simpler method, based on the GF_2ε_(λ) calculation formula, to compare the effects of different wavelengths on inactivation of microorganisms based on the required dose to achieve the goal of inactivation. For this, we read the log survival rate for each wavelength (Fig. 4B) and the dose of U280 required to achieve that log survival rate (Fig. 4A) to calculate the ratio of required dose to achieve a certain log survival rate. We named this evaluation method GF_4ε_(λ). To verify the validity of this calculation methods, we calculated the inactivation rates of irradiation with U254, U267, and U270 LEDs compared with that of the U280 LED. Then, we examined whether dose-response curves for U254, U267, and U270 LEDs could be fitted to that for the U280 LED. The results indicated that the dose-response curves of U254, U267, and U270 LEDs had values similar to that of the U280 LED (Fig. S3). Accordingly, we assessed the inactivation rate of each wavelength (250–365 nm) using this proposed calculation method (Fig. 4D). Although comparisons are typically made between conditions with different log survival rates as the reference when comparing multiple wavelengths other than the standard wavelength, our method enabled easy evaluation of the UV sensitivity of multiple wavelengths relative to the standard wavelength by using only the dose-dependent inactivation curve of standard wavelength and the inactivation curve for each wavelength at a constant dose.

## 4 Conclusions

**Fig. 5:**
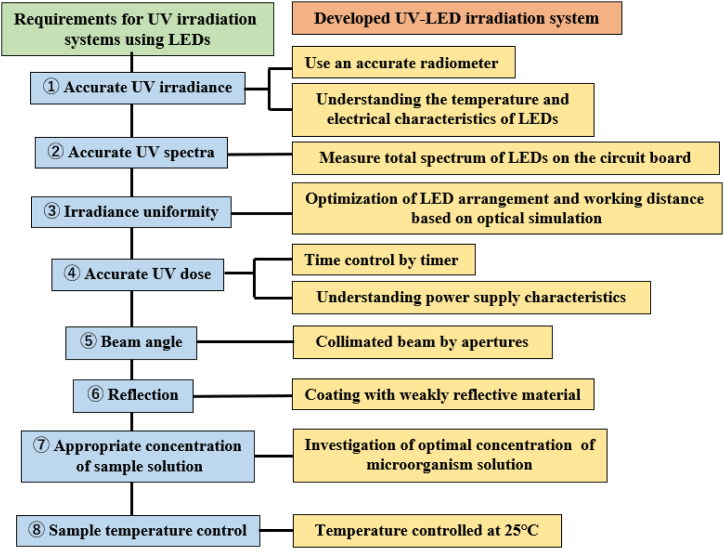
Requirements for UV irradiation systems (LEDs and device conditions).

In this study, we developed a UV light source system based on strict irradiation conditions and demonstrated the validity of the developed device as a standardized light source by evaluation of the effects of irradiation on inactivation of *E. coli* (Fig. 5). Using a comparison of the inactivation effect at each UV wavelength under uniform irradiation conditions with the U280 LED as the standard, we accurately evaluated the wavelength dependence of the inactivation effect from 250 to 300 nm, and confirmed that the 267 nm LED has a highest inactivation effect. Accurate measurement data for the UV sensitivity of each microorganism using this device may contribute to the establishment of food and water hygiene control methods and the development of equipment for the prevention of infectious diseases.

## Data availability statement

Data will be made available on request.

## CRediT authorship contribution statement

**Kai Ishida:** Conceptualization, Data curation, Formal analysis, Investigation, Methodology, Writing – original draft. **Yushi Onoda:** Conceptualization, Data curation, Formal analysis, Investigation, Methodology, Writing – original draft. **Yasuko Kadomura-Ishikawa:** Methodology, Validation, Investigation. **Miharu Nagahashi:** Investigation, Methodology. **Michiyo Yamashita:** Investigation, Methodology, Validation. **Shiho Fukushima:** Investigation, Methodology, Validation. **Toshihiko Aizawa:** Data curation, Formal analysis, Methodology, Resources. **Shigeharu Yamauchi:** Conceptualization, Funding acquisition, Investigation, Project administration, Resources, Supervision, Writing – review & editing. **Yasuo Fujikawa:** Investigation, Resources, Validation, Writing – review & editing. **Tomotake Tanaka:** Conceptualization, Funding acquisition, Project administration, Resources, Supervision. **Takashi Uebanso:** Writing – review & editing. **Masatake Akutagawa:** Investigation, Methodology. **Kazuaki Mawatari:** Conceptualization, Investigation, Methodology, Supervision, Writing – review & editing. **Akira Takahashi:** Conceptualization, Funding acquisition, Supervision, Visualization, Writing – review & editing.

## Declaration of competing interest

The authors declare that they have no known competing financial interests or personal relationships that could have appeared to influence the work reported in this paper.

## References

[1] Delogu G., Sali M., Fadda G. (2013). The biology of Mycobacterium tuberculosis infection. Mediterr J Hematol Infect Dis.

[2] Mutreja A., Kim D.W., Thomson N., Connor T.R., Lee J.H., Kariuki S., Croucher N.J., Choi S.Y., Harris S.R., Lebens M., Niyogi S.K., Kim E.J., Ramamurthy T., Chun J., Wood J.L.N., Clemens J.D., Czerkinsky C., Nair G.B., Holmgren J., Parkhill J., Dougan P. (2013). Evidence for multiple waves of global transmission within the seventh cholera pandemic. Nature.

[3] Egger N.B., Kainz K., Schulze A., Bauer M.A., Madeo F., Carmona-Gutierrez D. (2022). The rise of Candida auris: from unique traits to co-infection potential. Microb Cell.

[4] Najeeb H., Siddiqui S.A., Anas Z., Ali S.H., Usmani S.U.R., Jawed F., Jatoi H.N. (2022). The menace of Candida auris epidemic amidst the COVID-19 pandemic: a systematic review. Diseases.

[5] Michaelis M., Doerr H.W., Cinatl J. (2009). An influenza A H1N1 virus revival - pandemic H1N1/09 virus. Infection.

[6] Han S., Kumar P., Hossain I., Byun K.H., Choi C., Ha S.D. (2020). COVID-19 pandemic crisis and food safety Implications and inactivation strategies. Trends Food Sci Technol. Mar.

[7] Antunes P., Novais C., Peixe L. (2020). Food-to-humans bacterial transmission. Microbiol Spectr. Jan.

[8] Chen S.C., Chio C.P., Jou L.J., Liao C.M. (2009). Viral kinetics and exhaled droplet size affect indoor transmission dynamics of influenza infection. Indoor Air. Oct.

[9] Rodríguez-Lázaro D., Cook N., Ruggeri F.M., Sellwood J., Nasser A., Nascimento M.S.J., Agostino M.D., Santos R., Saiz J.C., Rzeżutka A., Bosch A., Gironés R., Carducci A., Muscillo M., Kovač K., Diez-Valcarce M., Vantarakis A., Bonsdorff C.H., Husman A.M.R., Hernández M., Poel W.H.M. (2012). Virus hazards from food, water and other contaminated environments. FEMS Microbiol. Rev..

[10] Köhler J.R., Hube B., Puccia R., Casadevall A., Perfect J.R. (2017). Fungi that infect humans. Microbiol Spectr. Jun.

[11] Wei J., Li Y. (2016). Airborne spread of infectious agents in the indoor environment. Am. J. Infect. Control.

[12] Mao Yi, Qiu Jianping, Zhang Peiqin, Fei Zhengxin, Bian Chaoqun, Jushi Janani Baadal, Ali Fakhri (2022). A strategy of silver Ferrite/Bismuth ferrite nano-hybrids synthesis for synergetic white-light photocatalysis, antibacterial systems and peroxidase-like activity. J. Photochem. Photobiol. Chem..

[13] Liu Yanni, Zhou Xu, Zhu Songlei, Ali Fakhri, Gupta Vinod Kumar (2022). Evaluation of synergistic effect of polyglycine functionalized gold/iron doped silver iodide for colorimetric detection, photocatalysis, drug delivery and bactericidal applications. J. Photochem. Photobiol. Chem..

[14] Liu Ye, Zong Lina, Zhang Chunxiao, Liu Wenjing, Ali Fakhri, Gupta Vinod Kumar (2021). Design and structural of Sm-doped SbFeO3 nanopowders and immobilized on poly (ethylene oxide) for efficient photocatalysis and hydrogen generation under visible light irradiation. Surface. Interfac..

[15] Bahadoran Ashkan, Liu Qinglei, Masudy-Panah Saeid, De Lile Jeffrey Roshan, Ramakrishna Seeram, Ali Fakhri, Gupta Vinod Kumar (2021). Assessment of silver doped cobalt titanate supported on chitosan-amylopectin nanocomposites in the photocatalysis performance under sunlight irradiation, and antimicrobial activity. Surface. Interfac..

[16] Chen Lian, Hosseini Mojgan, Ali Fakhri, Fazelian Nafiseh, Mohammadi Nasr Saeed, Nobakht Nastaran (2019). Synthesis and characterization of Cr2S3–Bi2O3 nanocomposites: photocatalytic, quenching, repeatability, and antibacterial performances. J. Mater. Sci. Mater. Med..

[17] Ali Fakhri, Naji Mahsa, Fatolahi Leila, Afshar Nejad Pedram (2017). Synthesis and characterization of Fe3O4 and CdTe quantum dots anchored SnO2 nanofibers and SnO2 nanospheres for degradation and removal of two carcinogen substance. J. Mater. Sci. Mater. Med..

[18] Zheng Zhi-Bo, Sun Jiang-Jie, Ali Fakhri, Surendar A., Aygul Z. (2018). Ibatova & Jia-Bao Liu. Synthesis, photocatalytic, optical, electronic and biological properties of the CoS2–CuS on cellulose nanocomposites as novel nano catalyst by a sonochemical technology. J. Mater. Sci. Mater. Med..

[19] Food and Drug Administration, HHS. (2000). https://www.federalregister.gov/documents/2000/11/29/00-30453/irradiation-in-the-production-processing-and-handling-of-food.

[20] Mamane-Gravetz H., Linden K.G., Cabaj A., Sommer R. (2005). Spectral sensitivity of Bacillus subtilis spores and MS2 coliphage for validation testing of ultraviolet reactors for water disinfection. Environ. Sci. Technol..

[21] Chen R.Z., Craik S.A., Bolton J.R. (2009). Comparison of the action spectra and relative DNA absorbance spectra of microorganisms: information important for the determination of germicidal fluence (UV dose) in an ultraviolet disinfection of water. Water Res. Dec.

[22] Bolton J.R., Beck S., Linden K.G. (2015). https://www.iuva.org/resources/Resource%20Documents/Bolton-Protocol%20for%20the%20Determination%20of%20Fluence.pdf.

[23] Beck S.E., Wright H.B., Hargy T.M., Larason T.C., Linden K.G. (2015). Action spectra for validation of pathogen disinfection in medium-pressure ultraviolet (UV) systems. Water Res. Mar.

[24] Beck S.E., Rodriguez R.A., Linden K.G., Hargy T.M., Larason T.C., Wright H.B. (2014). Wavelength dependent UV inactivation and DNA damage of adenovirus as measured by cell culture infectivity and long range quantitative PCR. Environ. Sci. Technol..

[25] Schuit M.A., Larason T.C., Krause M.L., Green B.M., Holland B.P., Wood S.P., Grantham S., Zong Y., Zarobila C.J., Freeburger D.L., Miller D.M., Bohannon J.K., Ratnesar-Shumate S.A., Blatchley E.R. (2020). SARS-CoV-2 inactivation by ultraviolet radiation and visible light is dependent on wavelength and sample matrix. Aug..

[26] Wan Q., Cao R., Wen G., Xu X., Xia Y., Wu G., Li Y., Wang J., Xu H., Lin Y., Huang T. (2022). Efficacy of UV-LED based advanced disinfection processes in the inactivation of waterborne fungal spores: kinetics, photoreactivation, mechanism and energy requirements. Sci. Total Environ..

[27] Nyangaresi P.O., Rathnayake T., Beck S.E. (2023). Evaluation of disinfection efficacy of single UV-C, and UV-A followed by UV-C LED irradiation on Escherichia coli, B. spizizenii and MS2 bacteriophage, in water. Sci. Total Environ..

[28] Sonntag F., Liu H., Neugart S. (2023). Nutritional and physiological effects of postharvest UV radiation on.

[29] Kheyrandish A., Mohseni M., Taghipour F. (2018). Protocol for determining ultraviolet light emitting diode (UV-LED) fluence for microbial inactivation studies. Environ. Sci. Technol..

[30] Bolton J.R., Mayor-smith I., Linden K.G. (2015). Rethinking the concepts of fluence (UV dose) and fluence rate: the importance of photon-based units. J. Photochem. Photobiol..

[31] Sun W., Jing Z., Zhao Z., Yin R., Santoro D., Mao T., Lu Z. (2023). Dose−Response behavior of pathogens and surrogate microorganisms across the ultraviolet-C spectrum: inactivation efficiencies, action spectra, and mechanisms. Environ. Sci. Technol..

[32] Nelson K.L., Boehm A.B., Davies-colley R.J., Dodd M.C., Kohn T., Linden K.G., Liu Y., Maraccini P.A., McNeill K., Mitch W.A., Nguyen T.H., Wigginton K.R., Zepp R.G. (2018). Sunlight-mediated inactivation of health-relevant microorganisms in water a review of mechanisms and modeling approaches. Environ Sci Process Impacts.

[33] Sancar A. (2003). Structure and function of DNA photolyase and cryptochrome blue-light photoreceptors. Chem Rev. Jun.

[34] Li G.Q., Wang W.L., Huo Z.Y., Lu Y., Hu H.Y. (2017). Comparison of UV-LED and low-pressure UV for water disinfection: photoreactivation and dark repair of Escherichia coli. Water Res..

[35] Le T.T.T., Mawatari K., Maetani M., Yamamoto T., Hayashida S., Iba H., Aihara M., Hirata A., Shimohata T., Uebanso T., Takahashi A. (2012). VP2118 has major roles in Vibrio parahaemolyticus response to oxidative stress. Biochim. Biophys. Acta.

[36] Bolton J.R. (2017). Action spectra: a review. IUVA News.

[37] Gates F.L. (1930). A study of the bacterial action of ultra III the absorption of ultra violet light by bacteria. J. Gen. Physiol..

[38] Sholtes K., Linden K.G. (2019). Pulsed and continuous light UV LED: microbial inactivation, electrical, and time efficiency. Water Res..

[39] Beck S.E., Ryu H., Boczek L.A., Cashdollar J.L., Jeanis K.M., Rosenblum J.S., Lawal O.R., Linden K.G. (2017). Evaluating UV-C LED disinfection performance and investigating potential dual-wavelength synergy. Water Res..

[40] Kumiko O., Surapong R., Mie M. (2019). Inactivation of health-related microorganisms in water using UV light-emitting diodes. Water Supply.

[41] Rattanakul S., Oguma K. (2018). Inactivation kinetics and efficiencies of UV-LEDs against Pseudomonas aeruginosa, Legionella pneumophila, and surrogate microorganisms. Water Res..

[42] Gustavsson T., Bányász Á., Lazzarotto E., Markovitsi D., Scalmani G., Frisch M.J., Barone V., Improta R. (2006). Singlet excited-state behavior of uracil and thymine in aqueous solution: a combined experimental and computational study of 11 uracil derivatives. J. Am. Chem. Soc..

[43] Mahsa M., Madjid M., James R.B. (2021). Sensitivity of bacteria, Protozoa, viruses, and other microorganisms to ultraviolet radiation. J Res Natl Inst Stand Technol. Jun 29.

[44] Xu L., Zhang C., Xu P., Wang X.C. (2018). Mechanisms of ultraviolet disinfection and chlorination of Escherichia coli: culturability, membrane permeability, metabolism, and genetic damage. J. Environ. Sci. (China).

