# Supplementary material for: Development of a standard evaluation method for microbial UV sensitivity using light-emitting diodes
Source: Heliyon. 2024 Mar 8;10(6):e27456. doi: 10.1016/j.heliyon.2024.e27456 (PMC10951525; doi:10.1016/j.heliyon.2024.e27456)
Supplement: Multimedia component 1 [file mmc1.docx]

Supporting Information

**Development of a Standard Evaluation Method for Microbial UV sensitivity Using Light-Emitting Diodes**

Kai Ishida^1‡^, Yushi Onoda^1,3‡^, Yasuko Kadomura-Ishikawa^1^, Miharu Nagahashi^2^, Michiyo Yamashita^2^, Shiho Fukushima^2^, Toshihiko Aizawa^3^, Shigeharu Yamauchi^3^, Yasuo Fujikawa^3^, Tomotake Tanaka^3^, Takashi Uebanso^1,2^, Masatake Akutagawa^4^, Kazuaki Mawatari^1,2^, Akira Takahashi^1,2*^

^1^Department of Microbial Control, Institute of Biomedical Sciences, Tokushima University Graduate School, Tokushima, Japan

^2^Department of Preventive Environment and Nutrition, Institute of Biomedical Sciences, Tokushima University Graduate School, Tokushima, Japan

^3^Nichia Corporation, Tokushima, Japan

^4^Department of Electrical and Electronic Engineering, Graduate School of Technology, Industrial and Social Sciences, University of Tokushima, Tokushima, Japan

*Corresponding author: Akira Takahashi, Ph.D. [akiratak@tokushima-u.ac.jp](mailto:akiratak@tokushima-u.ac.jp)

Tel: +81-88-633-9598, Fax: +81-88-633-7092

‡These authors are equal contributors to this work and designated as co-first authors.

Summary: 3 Figure

**Table of contents**

Figure S1 Cosine response characteristics of the radiometers……………………………………....S3

Figure S2 Characteristics of the UV-LED irradiation system……………………………………….S4

Figure S3 Dose response of inactivation of *E. coli* by the U280 LED and mapped dose responses from U254, U267, and U270 LEDs………………………………………………………………….S5

**Figures**

**
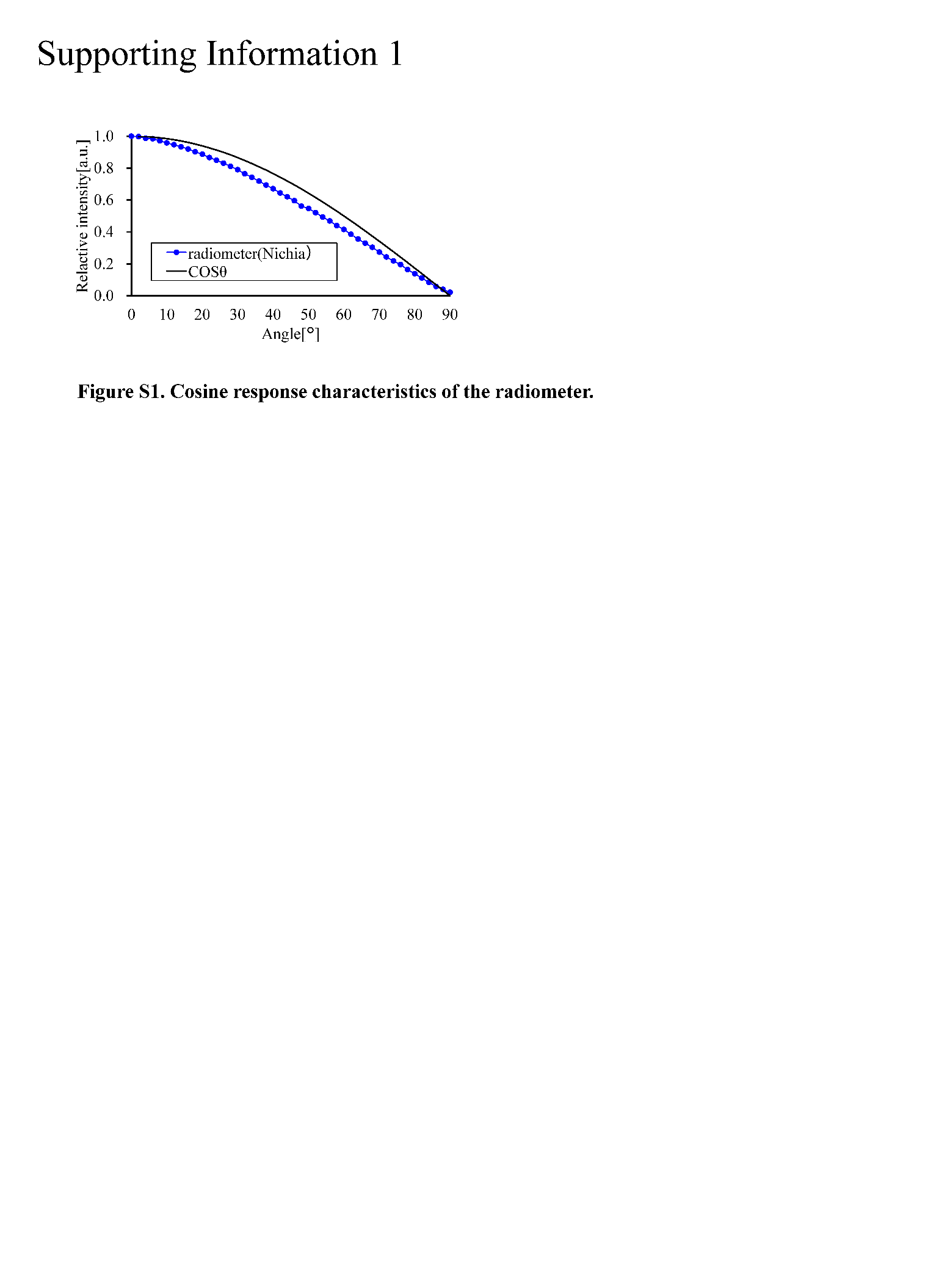
**

**Figure S1. Cosine response characteristics of the radiometer.**

**
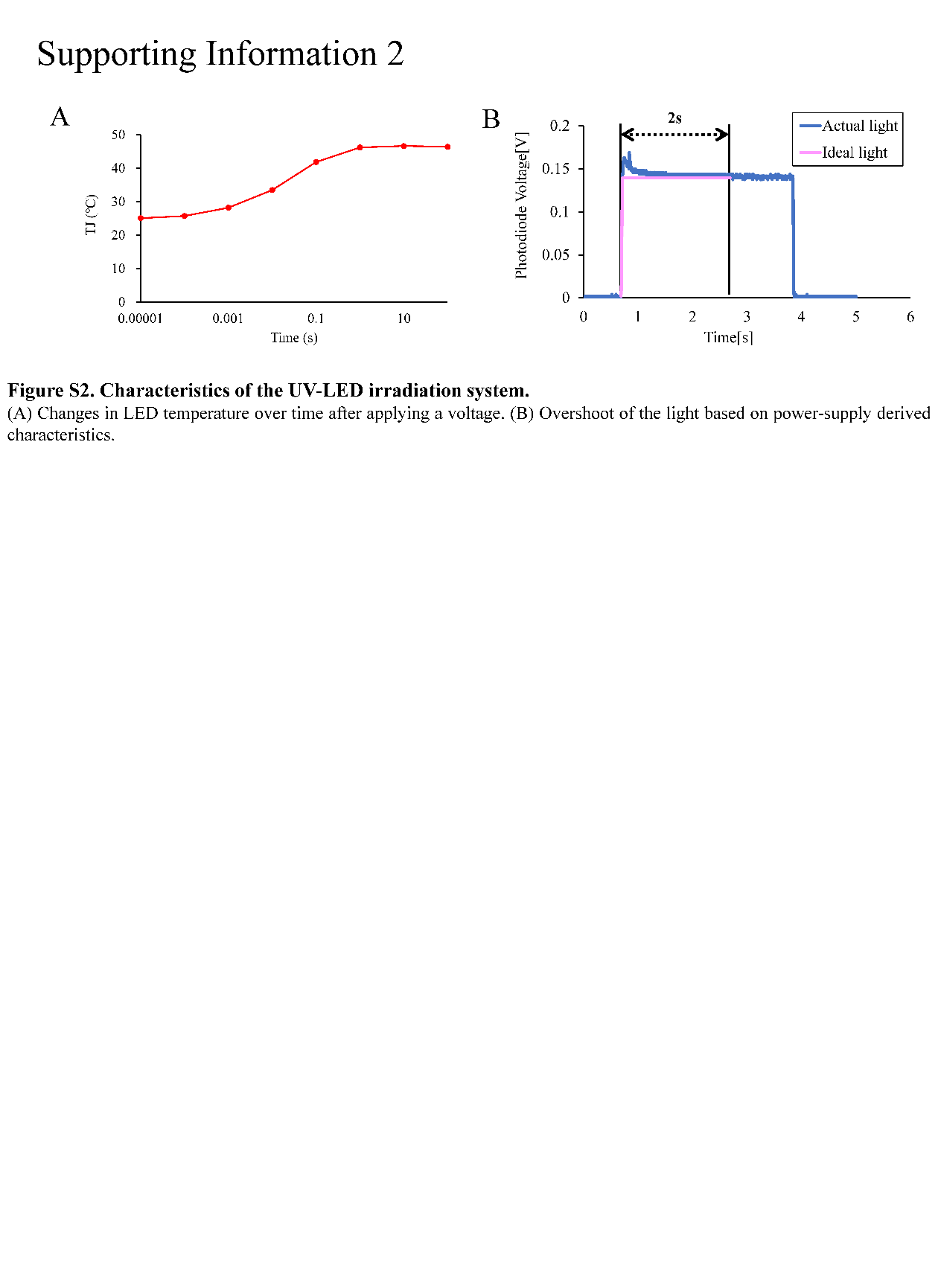
**

**Figure S2. Characteristics of the UV-LED irradiation system.**

(A) Changes in LED temperature over time after applying a voltage. (B) Overshoot of the light based on power-supply derived characteristics.

**
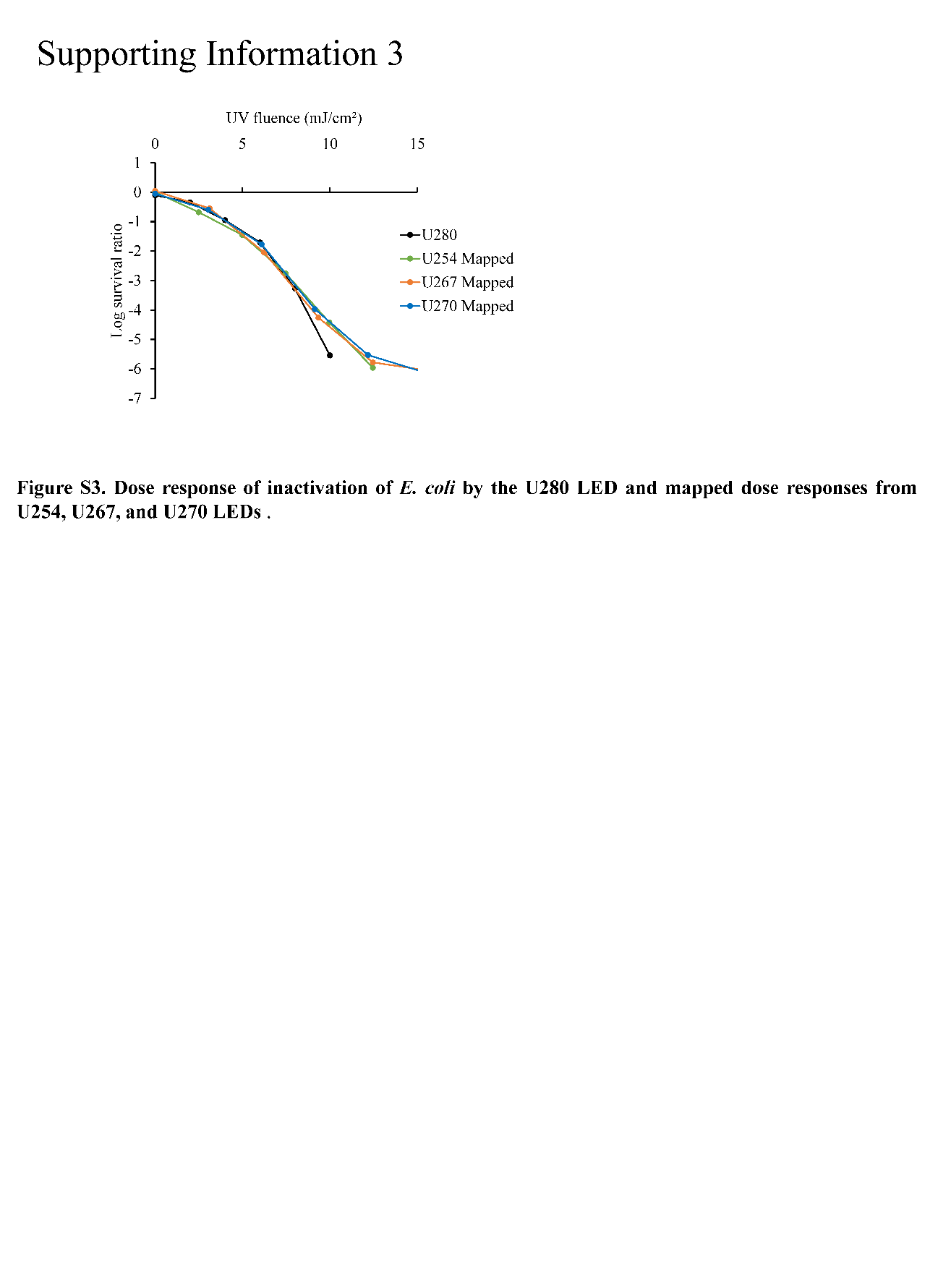
**

**Figure S3. Dose response of inactivation of E. coli by the U280 LED and mapped dose responses from U254, U267, and U270 LEDs.**
